# Development and Validation of Green UV Derivative Spectrophotometric Methods for Simultaneous Determination Metformin and Remogliflozin from Formulation: Evaluation of Greenness

**DOI:** 10.3390/ijerph18020448

**Published:** 2021-01-08

**Authors:** Mahesh Attimarad, Anroop B. Nair, Nagaraja Sreeharsha, Bandar E. Al-Dhubiab, Katharigatta N. Venugopala, Pottathil Shinu

**Affiliations:** 1 Department of Pharmaceutical Sciences, College of Clinical Pharmacy, King Faisal University, Al Hofuf 31982, Saudi Arabia; anair@kfu.edu.sa (A.B.N.); sharsha@kfu.edu.sa (N.S.); baldhubiab@kfu.edu.sa (B.E.A.-D.); kvenugopala@kfu.edu.sa (K.N.V.); 2 Department of Pharmaceutics, Vidya Siri College of Pharmacy, Bangalore 560035, India; 3 Department of Biotechnology and Food Technology, Durban University of Technology, Durban 4001, South Africa; 4 Department of Biomedical Sciences, College of Clinical Pharmacy, King Faisal University, Al Hofuf 31982, Saudi Arabia; spottathail@kfu.edu.sa

**Keywords:** remogliflozin, metformin, UV derivative spectroscopy, validation, formulation, ecofriendly

## Abstract

The recent trend in green analytical chemistry is the development of green analytical methods using environmentally friendly solvents. Therefore, three ecofriendly manipulated UV spectroscopic techniques have been validated for the concurrent quantification of newly approved remogliflozin etabonate (REM) and metformin HCl (MET) tablets using water as a solvent. The first method was established using first derivative absorption spectroscopic method by determining the peak amplitude at 233.0 nm for REM and 252.2 nm for MET, a zero crossing of one the component. The second and third methods were based on the peak amplitude difference and first-order derivative absorption of the ratio spectra developed by the manipulation of scanned UV spectra. REM and MET showed good linearity in the series of 1–20 µg ml^−1^ and 2.5–35 µg ml^−1^, respectively, by all three methods with an excellent correlation coefficient (r^2^ ≥ 0.998). Further, the proposed UV spectroscopic techniques were validated as per International Council for Harmonization guidelines. The methods showed good sensitivity, accuracy, and precision. Anticipated procedures were effectively utilized for the concurrent quantification of REM and MET in laboratory prepared mixtures and tablets. The high percent recovery with low standard deviation found for both analytes by all three methods confirms the accuracy and precision of the procedures. Finally, the greenness of the proposed spectroscopic methods, evaluated by semi-quantitative and quantitative methods, showed the eco-friendly nature of the methods. Furthermore, the proposed approaches were simple, accurate, sensitive, economic, and environmentally friendly and hence can be utilized for regular quality control of REM and MET formulation.

## 1. Introduction

**Figure 1:**
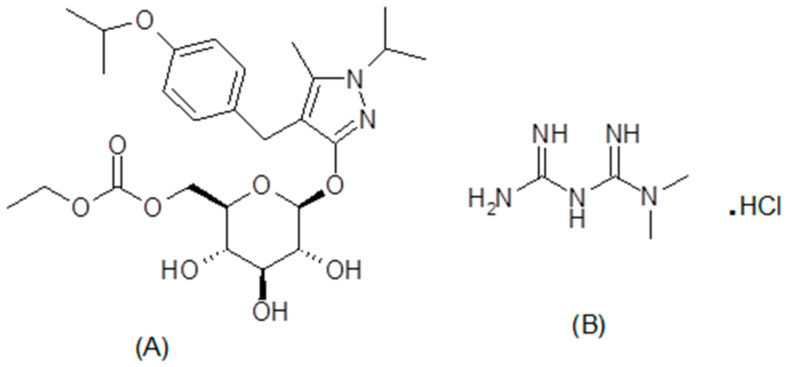
Chemical structure of remogliflozin etabonate (**A**) and metformin HCl (**B**).

Common endocrinological diseases, like Diabetes Mellitus Type 2 (DMT2), are already enormous and growing at alarming percentile throughout the world. As estimated, the number of diabetic patients will increase to more than 500 million by the year 2030 and to more than 700 million by 2045 [1,2]. Numerous studies have been undertaken to elucidate more efficacious antidiabetic drugs with improved glycemic control, with fewer adverse effects [3]. Sodium-glucose cotransporter-2 inhibitors (SGLT-2) were recently approved for the management of DMT2, [4,5] alone or in combination with other antidiabetic drugs. Remogliflozin etabonate (REM, Figure 1A) is a recent addition to the SGLT-2 class of antidiabetics [6,7] having better glycemic control along with added advantages such as insulin-independent action, reduction in body weight, controlling blood pressure, and its protective effect on the cardiovascular and renal system. REM acts by inhibiting the SGLT-2 enzyme responsible for reabsorption of sugar from the glomerular filtrate at convoluted part of nephron, thereby increasing the elimination of sugar in the urine (Glycosuria) and reducing the blood glucose level [5,6,7,8,9,10,11,12]. Metformin HCl (MET; Figure 1B) an oral biguanide antidiabetic drug is a preferred antidiabetic agent used for the management of DMT2 due to its safety and several advantages over other agents [13,14,15]. Hence, recently a solid dosage form consisting of metformin and remogliflozin has been approved by the Food and Drug Administration for DMT2. This combination acts through a different mechanism, such as a decrease in the release of glucose in liver and increase excretion of sugar in urine, hence, providing better glycemic control [16]. Further, combination showed protecting effect on the cardiovascular system by reducing the blood pressure and body mass index. Therefore, this makes the combination a better choice for the treatment of DMT-2 patients with cardiovascular comorbid states. 

Only two analytical methods [17,18] are described in the literature for the estimation of remogliflozin alone. Ankita et al. [17] established a zero-order UV-spectroscopic technique for the estimation of REM in pharmaceutical preparations, whereas Sigafoos et al. [18] utilized LC/MS method for estimation of REM from blood samples. Several methods are illustrated in the literature for the analysis of metformin alone and along with different classes of agents from formulations and body fluids. Recent methods reported were UV-spectrophotometry [19,20,21], RP-HPLC [22,23,24,25], HPTLC [24,25], capillary electrophoresis [26,27] and LCMS [28,29,30,31,32]. There were few reports in the literature for the simultaneous estimation of metformin along with SGLT-2 inhibitors [32,33]. However, UPLC [34], HPLC and UV second derivative spectroscopic methods [35] have been reported for the concurrent determination of metformin and remogliflozin. However, UPLC and HPLC methods requires sophisticated expensive instruments and toxic solvents such acetonitrile and methanol as mobile phase. Furthermore, no internal standard has been used in the reported UPLC method. Hence, current study describes the simple, rapid, eco-friendly UV derivative spectrophotometry method for concurrent determination of metformin HCl and remogliflozin etabonate. Further, the reported methods were validated and utilized for the concurrent quantification of both analytes from laboratory prepared solutions and pharmaceutical preparations. 

UV-spectroscopic methods are simple, rapid, and economical analytical methods for quality control of pharmaceutical preparations used regularly. However, direct UV spectroscopic methods suffer from multicomponent formulations effects and formulation additives effects [36,37]. Further, UV spectra REM and MET showed complete overlap, making it difficult to utilize direct UV-spectrophotometry for simultaneous determination of this combination. Hence, derivative and ratio derivative UV spectroscopic methods [36,37,38,39,40] were tried to resolve the multicomponent and additive effects, which were found suitable for the simultaneous determination of REM and MET without interference. Further, the proposed procedures were cost-effective and ecofriendly, as water was used as a major solvent throughout the experiments.

## 2. Materials and Methods

### 2.1. Chemicals and Reagents

Reference standards of REM (purity 99.2%) and MET (purity 98.96%) were purchased from Bioteck India Limited, Hyderabad, India. Ethanol used for the preparation of standard solution was analytical grade and has been procured from Sigma Aldrich, St. Louis, MO, USA. Ultra-pure water used during the experiments was arranged in the laboratory with Milli Q (Millipore USA) water purification system. REM (100 mg) and MET (500 mg/1000 mg) tablets were not available in the resident market; hence, tablets were prepared by mixing the required quantity of REM, MET, mannitol, talc, magnesium stearate, microcrystalline cellulose, colloidal silicon dioxide, and polyvinyl pyrrolidone. 

### 2.2. Instrument and Software

Shimadzu UV Spectrophotometer (UV-1700) (Tokyo, Japan) attached to the computer was utilized for recording the UV spectra. Quartz cuvettes with path length 1 cm were used for recording the spectra for both blank and sample. Instrument scanning speed was adjusted to medium speed with a slit width of 2 nm. UV-probe version 2.0 software (Shimadzu, Kyoto, Japan) provided with the Shimadzu UV Spectrophotometer was used for the processing of the recorded spectra. Manipulation of UV spectra displayed noise; hence, to generate the smooth spectra, 4 nm has been used as a smoothing wavelength. Graduated long neck flasks used for preparing the stock solutions, working standard and formulation solutions were calibrated before use.

### 2.3. Preparation of Standard Solutions

Primary standard solutions were prepared by transferring pure drug REM (50 mg) in 50 mL ethanol, because REM was slightly soluble in water, whereas MET (50 mg) was dissolved in 50 mL ultrapure water as metformin HCl is highly water-soluble. Further, working calibration solutions were organized from the primary standards with ultrapure water by maintaining the equivalent amount of ethanol (200 µL) in all flasks. The blank solution consists of the same amount of ethanol (200 µL) as in the standard solutions and ultrapure water. 

### 2.4. Preparation of Sample Solutions and Laboratory Mixed Solutions

REM and MET tablets were unavailable in the resident market; hence, simulated tablets consisting of 100 mg REM along with 500 mg/1000 mg MET were prepared in the laboratory. Accurately weighed REM (200 mg) was mixed with MET (1000 mg) and MET (2000 mg) separately along with known amount of excipients like mannitol, talc, magnesium stearate, microcrystalline cellulose, colloidal silicon dioxide, and polyvinyl pyrrolidone. All ingredients were thoroughly mixed and the sufficient amount of mixture equivalent to 100 mg REM and 500 mg MET/1000 mg MET was conveyed to a 50 mL graduated flask consisting of 25 mL of ethanol separately. After sonication of flasks for 5 min, the mixture was transferred into other graduated flasks using Whatman filter paper. The filter paper was rinsed with ethanol, and lastly, the volume was attuned to 50 mL by adding ethanol. Further, sufficient amount of water has been added to the aliquot of the above solutions to get the quantity of analytes in the linearity range. The laboratory prepared solutions of diverse portions of REM and MET were organized by transferring the required quantity of REM and MET primary solutions into 10 mL volumetric flasks. 

### 2.5. Procedure 

#### 2.5.1. First Derivative Spectroscopic Method (FDS) 

Calibration curves were constructed separately by transferring an adequate volume of stock solution of REM and MET to 10 mL graduated flasks. The final concentration of solutions for calibration curve were 1 to 20 µg ml^−1^ for REM (1, 2, 4, 8, 12, 16, and 20 µg mL^−1^) and 2.5 to 35 µg mL^−1^ for MET (2.5, 5, 10, 15, 20, 25, 30, and 35 µg mL^−1^). UV absorption of solutions was recorded between wavelength 200 nm to 300 nm against a blank solution. Then the first–order derivative spectra were generated from the stored spectra through 4 nm as ∆λ along with an amplifying feature of 10. The peak amplitude of REM spectra at a wavelength of 233.0 nm and zero crossings for MET and peak amplitude of MET at a wavelength of 252.2 nm and zero crossings for REM were measured. Then the regression equations and correlation coefficients were computed from the linearity curves generated by drawing the graphs using these amplitudes against respective concentrations of REM and MET separately. 

#### 2.5.2. Ratio Absorbance Difference Method (RAD)

The appropriate volumes of REM and MET stock solutions were added into a 10 mL graduated flask separately to prepare 20 µg mL^−1^ and 10 µg mL^−1^ solutions respectively. UV absorptions of both solutions were measured between 200 nm and 300 nm and the spectra were saved in the computer. Furthermore, required amount of REM and MET stock solution were transferred into same 10 mL graduated long neck flasks to obtain 1 to 20 µg mL^−1^ of REM and 2.5 to 35 µg mL^−1^ MET. Then the UV absorption of the solutions was measured in the wavelength 200 to 300 nm, and the spectra were stored. For quantification of REM, ratio spectra were generated by divining the above mixture spectra by spectrum of MET (10 µg mL^−1^), which were smoothened with 4 nm and stored. The peak amplitude at 248.6 nm was subtracted from the peak amplitude at 277.8 nm of the ratio spectrum. Then the regression equations and correlation coefficients were computed from the linearity curves generated by drawing graphs using amplitude difference value and the corresponding amount of REM. Correspondingly, the ratio spectra of MET were generated by dividing the spectra of analyte mixtures with a spectrum of REM 20 µg mL^−1^. Further, the variance among the two peak amplitudes of the smoothened ratio spectra was computed at 242.4 and 221.2 nm, and linearity curves were built against the respective concentration of MET. 

#### 2.5.3. Ratio First Derivative Method (RFD)

Above recorded ratio spectra for REM were changed to first-order derivative spectra via 4 nm as ∆λ and peak amplitude was recorded at 288.4 nm. The regression equations and correlation coefficients were computed from the linearity curve raised by outlining a graph between peak amplitude and the corresponding concentration of REM. Similarly, ratio spectra of MET were changed to a first-order derivative ratio spectra by employing 4 nm as ∆λ. The spectrum height was recorded at 251.7 nm form the different concentrations spectra and plotted against the respective concentration of MET to generate the calibration curve. 

### 2.6. Application of Spectroscopic Methods to Formulations and Laboratory Mixed Solutions

A sufficient amount of sample solutions were transferred from the formulation solutions prepared using REM 100 mg and MET 500 mg into 10 mL graduated flasks to get the quantity of analytes as in the formulation ratio 2:10 µg mL^−1^ and 4:20 µg mL^−1^ correspondingly. Similarly, sample solutions were prepared using the formulation solutions prepared by REM 100 mg and MET 1000 mg to get the finishing amount of 2:20 µg mL^−1^ and 3:30 µg mL^−1^ of REM and MET, respectively. Similarly, laboratory mixed solutions were arranged by my transferring necessary volume of REM and MET stock solutions to get different ratios of both the analytes. Absorption of these solutions was measured in the UV wavelength 200 to 300 nm, and spectra were deposited in the workstation. For the first derivative absorption method above, recorded spectra were changed to first-order spectra using 4 nm as ∆λ. Peak amplitudes were noted at 233.0 nm for REM and 252.2 nm for MET. For the ratio derivative absorption method, the spectra were divided separately by MET (10 µg mL^−1^) and by REM (20 µg mL^−1^) spectra to get respective ratio derivative spectra. For REM, the peak amplitude difference between 277.8 and 248.6 nm was recorded form REM ratio spectra; similarly, for MET, the peak amplitude of ratio derivative spectra of MET at 242.4 and 221.2 nm was recorded. Further, for the first derivative ratio spectra, the above-computed ratio spectra were changed into first-order derivative ratio spectra through 4 nm as ∆λ. Then, the spectrum amplitude was recorded at 288.4 nm for REM and at 251.7 nm for MET. Finally, the amount of analytes was computed from the respective regression equations. 

## 3. Results and Discussion 

**Figure 2:**
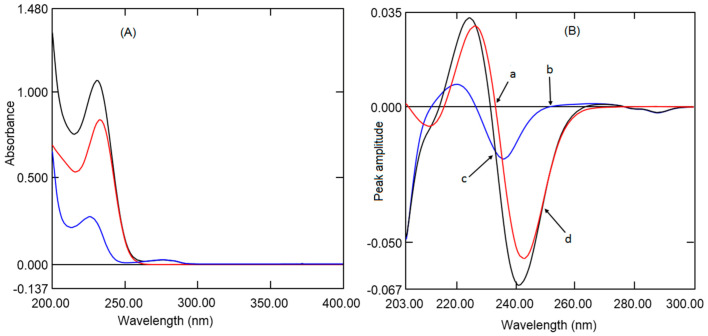
Normal spectra of remogliflozin (REM) (Blue), metformin (MET) (Red), and Mixture (Black) (**A**). First derivative spectra of REM, MET, and Mixture (**B**). Zero crossings of MET (a) and REM (b). Pure MET and mixture showing the same absorbance (c). Pure REM and Mixture showing the same absorbance (d).

UV spectrophotometric methods are extensively used for routine analysis of pharmaceutical preparations due to their simple, fast, economical, and precise results. However, multicomponent formulations having overlapping UV absorption make it difficult to analyze all analytes without prior separation. MET has UV absorption in the wavelength 200 to 260 nm with λ_max_ 232.9 nm, and it is completely overlapped with the UV absorption of REM, (Figure 2A) which has two λ_max_ at 229.9 and 276.6 nm. However, only REM has some absorption at 276.6 nm where MET has zero absorption. However, the intensity of absorption at 276.6 nm is very low; hence, absorption at this wavelength cannot be used for quantitative purposes. Therefore, derivative, ratio absorbance difference and ratio derivative methods were utilized to overcome the overlapping effect and effectively quantify both analytes in the presence of each other. Furthermore, both analytes did not show an absorbance above 300 nm; hence, UV scanning was performed in the wavelength 200 to 300 nm.

### 3.1. First Derivative Spectroscopic Method (FDS)

**Figure 3:**
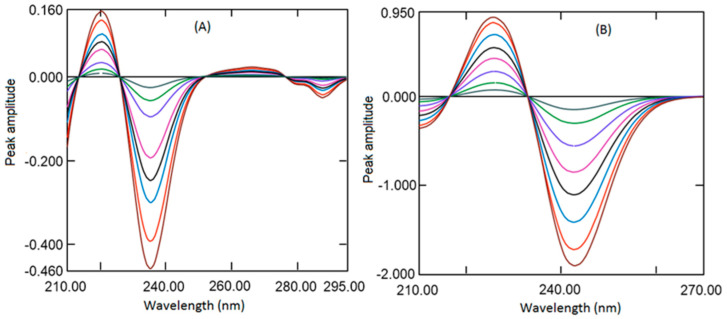
First derivative spectra of REM 1, 2, 4, 8, 12, 16, and 20 µg mL^−1^ (**A**), and MET 2.5, 5, 10, 20, 25, 30, and 35 µg mL^−1^ (**B**).

It is well established that the derivatization of UV absorption spectra increases the specificity and selectivity of analytes in multicomponent formulations by increasing the resolution. Derivatization also permits us to quantify one analyte co-existing with another analyte and eliminates the effects of excipients. In addition, the selection of wavelengths of derivative spectra of one of the analytes at the zero crossings of another analyte eliminates the interference from each other. [36,37] Hence, in the present work, first derivative absorption at zero crossings wavelength was developed for the quantification of analytes. Further, the fixed-dose combination of REM and MET is available as 100 mg with 500 mg/1000 mg respectively. The low amount of REM compared to MET showed very low absorption with first derivative spectra; hence, to increase the absorption at low concentration scaling, factor 10 was selected during derivatization. In the present work, UV absorption spectra for REM and MET were recorded and changed to first-order derivative spectra by means of 4 nm as ∆λ. Diverse wavelengths (1, 2, 4, and 8 nm) were tried as ∆λ, although 4 nm with a scaling factor of 10 showed better sensitivity. Therefore, 4nm as ∆λ with scaling factor 10 was selected for derivatization both analytes. First derivative spectrum REM (Figure 2B) exhibited two maxima at 220.4 and 266.2 nm and two minima at −235.3 and −287.7 nm. However, at 216.4, −232.9 and 287.7 nm, MET spectrum was zero crossings where REM had some absorption. However, at 216.4 and 287.7 nm, peak amplitude was low, whereas at 232.9 nm, peak amplitude was better along with good linearity. First derivative spectrum of MET (Figure 2B) showed one maximum at 225.4 nm and one minimum at −242.8 nm. Whereas at 213.5, 226.0and −252.2 nm REM spectrum showed zero crossings where MET had some absorption. Although at 213.5 nm the peak amplitude was low, also recovery from the formulation was not accurate, and at 226.0 nm, amplitude was good, but linearity was not appropriate. Wavelength 252.2 nm showed better amplitude along with a wide range of linearity. Hence, in the present work, different concentrations of REM (1 to 20 µg mL^−1^) and MET (2.5 to 35 µg mL^−1^) were scanned and converted to first derivative spectra, and peak amplitudes were measured at wavelength −232.9 nm and −252.2 nm for REM and MET respectively (Figure 3A,B). 

### 3.2. Ratio Absorbance Difference Method

The ratio spectroscopic method was based on dividing the mixture spectrum by one of the analyte spectra to obtain the ratio spectrum, which is free from the interference from the divisor analyte and excipients. Further, use of optimized spectrum as divisor reduces the noise and experimental errors. Another advantage of ratio spectra method over the zero crossing derivative method is that measurements were made in correspondence to the peaks; hence, the measurements are more accurate, sensitive, and specific. One of the disadvantages of zero crossing method is that a small drift at the zero crossing points will not coincide with the working wavelengths, making the measurements less accurate and precise. Hence, the ratio spectroscopic methods [34] were developed. The UV absorption of a mixture (A_RM_) of two components (REM and MET) is expressed as a sum of the product of the molar extinction coefficient and concentration. Given by the Beer’s law (Equation (1))

A_RM_ = Є_R_C_R_ + Є_M_C_M_ (1)

where, Є_R_ and Є_M_ are molar extinction coefficient of REM (R) and MET (M), respectively, at one particular wavelength, whereas C_R_ and C_M_ are the concentration of REM and MET, respectively. For quantification of one of the components from the mixture, absorption of only one component needs to be determined by canceling the absorption of another component, which can be carried out by dividing the absorption equation of combination by absorption equation of one of the analytes at a concentration _M˚_(A_M˚_ = Є_M_C_M˚_); the simplified Equation is (2)

A_RM_/A_M˚_ = A_R_/A_M˚_ + C_M_/C_M˚_ (2)

The C_M_/C_M˚_ is constant (K), the above equation can be simplified to Equation (3)

S_A_ = S_R_ + K (3)

where S_A_ is A_RM_/A_M˚_ absorbance of ratio spectrum of a mixture to one of the components at some concentration (_M˚_), S_R_ is the A_R_/A_M˚_ absorbance spectrum of one constituent REM to another constituent MET.

From the above equation, K could be excluded by computing the absorbance variance (∆S) at two separate wavelength points (λ1 and λ2) of spectra, conferring to the below equation

∆S = S_A1_ − S_A2_ = (S_R1_ + K) − (S_R2_ + K) = S_R1_ − S_R2_ (4)

where S _R1_ and S _R2_ were absorption values of spectra at wavelengths λ1 and λ2.

It is clear from the Equation (4) that, component M is excluded completely; hence, the absorbance calculated between peak amplitudes at two different points represents only one component R. The concentration of compound R in the mixture can be determined by creating the linearity curve by plotting the graph between peak amplitude differences of ratio spectra with the corresponding concentration of component R. Similar procedure can be adapted to determine another component M from the mixture. 

**Figure 4:**
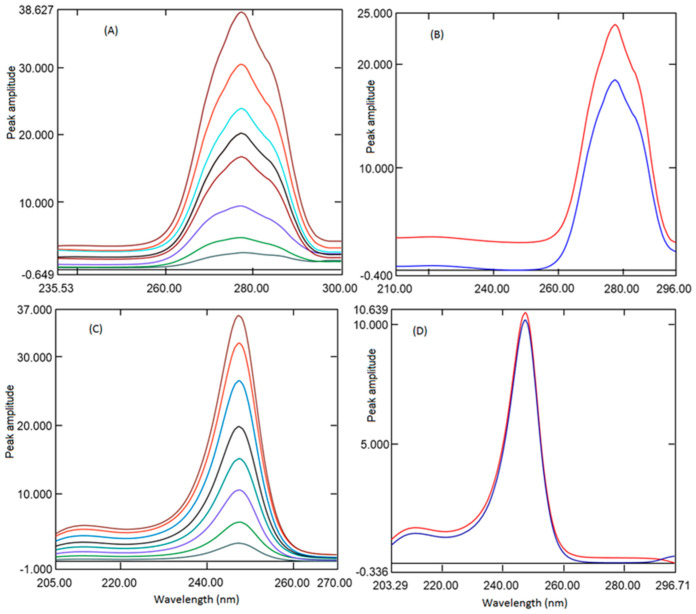
Ratio spectra of REM (1, 2, 4, 8, 10, 12, 16, and 20 µg mL^−1^) when 10 µg ml-1 MET was used as the divisor (**A**). Ratio spectra of standard pure REM and Mixture solution containing the same amount of REM (**B**) using 10 µg mL^−1^ MET as a divisor. Ratio spectra of MET (2.5, 5, 10, 15, 20, 25, 30, and 35 µg mL^−1^) when 20 µg mL^−1^ REM was used as the divisor (**C**). Ratio spectra of standard pure MET and Mixture solution containing the same amount of MET (**D**) using 20 µg mL^−1^ REM as a divisor.

In the current study, a mixture of REM and MET having varying concentrations has been scanned in the wavelength 200 to 300 nm. For determining the REM, ratio spectra were generated amongst REM spectra to the spectrum of MET (10 µg mL^−1^), and the peak heights difference were determined from the ratio spectra (Figure 4A) at 277.8 and 248.6 nm, which represents the concentration of REM only. Figure 4B showed the ratio absorption spectra of a mixture of REM and MET and standard REM consisting of the same amount of REM. The difference in the amplitude is constant (C_M_/C_M˚_), which could be omitted by deducting the peak amplitude at 248.6 nm from 277.8 nm. The ratio spectra generated from mixture spectra and pure standard REM spectra showed the same amplitude difference, indicating REM could be estimated even in mixture with MET. Similarly, MET has been quantified by generating the ratio spectra by dividing the mixture spectra by REM spectra (20 µg mL^−1^). The two wavelengths selected were peak 247.4 nm and trough 221.2 nm; the peak amplitude difference between these two points is correlated to the amount of MET (Figure 4C). The linearity curve was generated by determining the peak amplitude difference between 247.4 and trough 221.2 nm and plotting against the respective concentration of MET. Figure 4D showed the difference between the two points is same for spectra generated from the mixture spectrum and pure standard MET spectrum consisting of the same amount of MET. This demonstrated the application of absorbance difference method for analysis of MET in presence of REM.

### 3.3. Ratio First Derivative Method (RFD)

The impact of constant (C_M_/C_M˚_), present in the Equation (2), could be removed by transferring the ratio spectrum into first-order derivative spectrum. Derivative spectrum gives a number of positives (maximum) and negatives (minimum) spectra points; the amplitude of maximum or minimum is proportional to one of the components, eliminating the effect of another analyte as well as that of formulation excipients. Furthermore, the major advantage of ratio derivative spectroscopic method is measurements can be made at any of the maximum or minimum peaks, no need for working only at zero crossing points as in the zero-crossing derivative spectroscopic methods. The derivatization of ratio spectra gives high amplitude values when compared to derivatization of normal spectra. Further, this is a substitute to the aforementioned first derivative spectroscopic and ratio absorbance difference method for quantification of REM in presence of MET and Vice versa.

**Figure 5:**
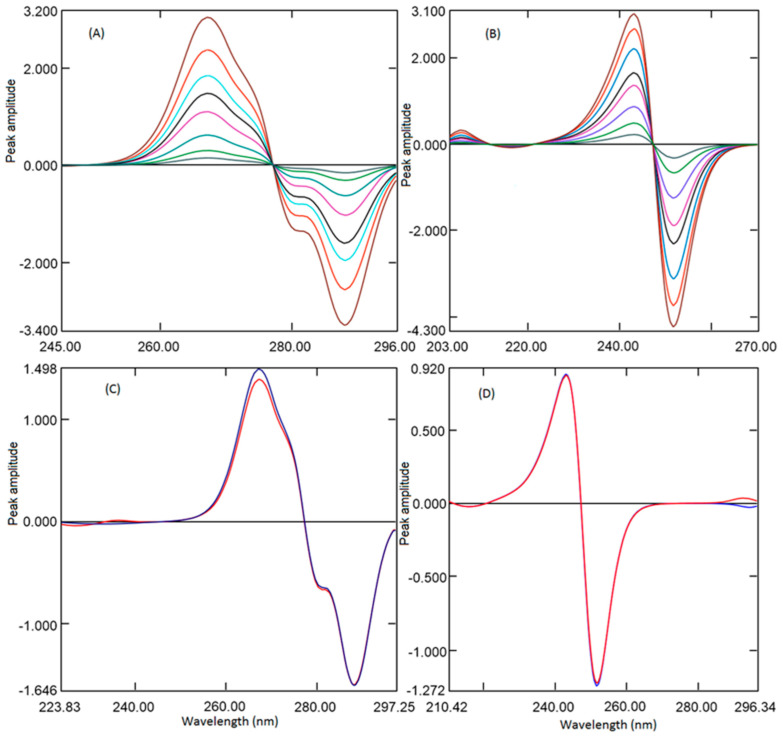
Ratio first derivative spectra of REM (1, 2, 4, 8, 10, 12, 16, and 20 µg mL^−1^) when 10 µg mL^−1^ MET was used as the divisor (**A**) and MET (2.5, 5, 10, 15, 20, 25, 30, and 35 µg mL^−1^) when 20 µg mL^−1^ REM was used as the divisor (**B**). Ratio first-order derivative spectra of standard pure REM and in combination with MET containing the same amount of analytes (**C**) using 10 µg mL^−1^ MET as a divisor. Ratio first spectra of standard pure MET and in combination with REM containing the same amount of analytes (**D**) using 20 µg mL^−1^ REM as a divisor.

Therefore, the above-generated spectra were transmuted to first-order derivative ratio spectra utilizing divider wavelength of 4 nm. Diverse wavelengths between 1 and 8 nm were envisaged as ∆λ; the spectra generated with 4 nm exhibited better sensitivity, indicating why 4 nm was used. The first-order derivative spectra generated for REM displayed one positive peak at 267.2 nm and two negative peaks at −280.0 and −288.4 nm. (Figure 5A) However, at all wavelengths, amplitude was good, but at −288.4, percentage recovery and linearity were better; hence, −288.4 nm was selected for further study. The first derivative spectra of MET presented one maximum at 243.0 nm and one minimum at −251.7 nm (Figure 5B). The peak amplitude was good at both wavelengths; however, good recovery and good sensitivity were observed at 251.7 nm; hence, 251.7 nm was utilized for construction of linearity curve. Ratio of first-order derivative spectra generated from the mixture (REM and MET) and a pure standard (REM Or MET) were compared in Figure 5C,D. The amplitudes showed by analyte were found to be the same in the mixture and pure analyte spectra consisting of the same amount of analytes.

### 3.4. Method Validation 

Validity of all the suggested spectroscopic methods was performed according to the ICH guidelines for linearity, the limit of determination and quantification, accuracy, precision, selectivity, and stability of analytes in the experimental and storage conditions.

#### 3.4.1. Linearity

As per the Beer’s Law, the good linearity was achieved in the range considering the ratio of analytes in the pharmaceutical preparation with a good correlation coefficient. Further, the intercepts of the calibration curves were found to be not significant. For constructing of calibration curves (Figure S1) series of eight solutions consisting of 1 to 20 µg mL^−1^ of REM (1, 2, 4, 8, 10, 12, 16, and 20 µg mL^−1^) and 2.5 to 35 µg mL^−1^ of MET (2.5, 5, 10, 15, 20, 25, 30, and 35 µg mL^−1^) were analyzed in triplicate. All experiments were conducted as per the optimized condition, and the regression parameters were tabulated (Table 1). 

#### 3.4.2. Limit of Determination and Quantification

Limit of determination and quantification shows the sensitivity of the analytical methods, which was performed according to the following Equation (5):

LOD = 3.3 σ/δ and LOQ = 10 σ/δ(5)

where σ symbolizes the standard deviation of the y-intercepts of regression line, and δ is the slope of the calibration curves. The computed LOD and LOQ were represented in Table 1. The standard solutions of REM and MET were prepared and analyzed at LOD and LOQ concentration level by all three methods, and the results were in agreement with the concentration of the solutions. The low LOD and LOQ values of both analytes in general and REM in particular were good enough to analyze the analytes in the formulation even with a high difference in the concentration of both analytes. 

#### 3.4.3. Accuracy and Precision 

Both intra-day and inter-day accuracy and precision were determined using four different concentrations covering the complete calibration range. Solutions consisting of 2.5, 7.5, 12.5, and 17.5 µg mL^−1^ of REM and 4, 14, 24, and 32 µg mL^−1^ of MET were prepared and analyzed in triplicate. For intra-day, solutions were analyzed in one day, and for inter-day, similar solutions were evaluated for three consecutive days (Figure S2). Concentration was determined by all three methods using corresponding regression equations. Accuracy was represented as percent recovery, which was found to be in the range of 98.4% to 101.5% whereas precision was expressed as percent relative standard deviation and was within 2%. (Table 2). 

#### 3.4.4. Selectivity

Laboratory mixed solutions were analyzed by the proposed spectroscopic methods to assess the selectivity (Figure S3). Diverse ratios of REM and MET (below and above the formulation concentration ratio) were prepared in the linearity range and evaluated in triplicate. The assay results, (Table 3) found to be in the range of 98.16% to 101.37% for REM and 98.10% to 101.76% for MET, confirming the selectivity of the analytical procedures in analyzing the dissimilar ratios of both drugs. Furthermore, results indicated that formulations available in different ratios of REM and MET could be analyzed with high accuracy along with good precision. 

#### 3.4.5. Stability Studies

Stability studies were performed by keeping the standard solutions at ambient temperature for 24 h and in the refrigerator at 6 °C for 7 days. The assay results of samples were compared between the first day, with the results after 24 h and 7 days. No significant dissimilarity was witnessed in the assay results, indicating the stability of analytes at room temperature and refrigerator temperature for more than 24 h and 7 days, respectively. 

#### 3.4.6. Determination of REM and MET in the Formulation and Standard Addition Method

REM and MET formulations, available in two different fixed-dose ratios (100 mg of REM and 500/1000 mg of MET) were analyzed using two concentrations in each ratio. Further, percent recoveries were calculated by standard addition method at three different concentrations by adding 50%, 100%, and 150% to earlier evaluated tablet solution comprising of 2 µg mL^−1^ of REM and 10 µg mL^−1^ of MET (Figure S4). The assay results (Table 4) of the formulation were in agreement with the actual amount of both analytes. Further, the percent recovery was found to be well within the standard range, demonstrating the absence of interference from the formulation excipients. 

### 3.5. Greenness Evaluation of the Proposed Methods

**Figure 6:**
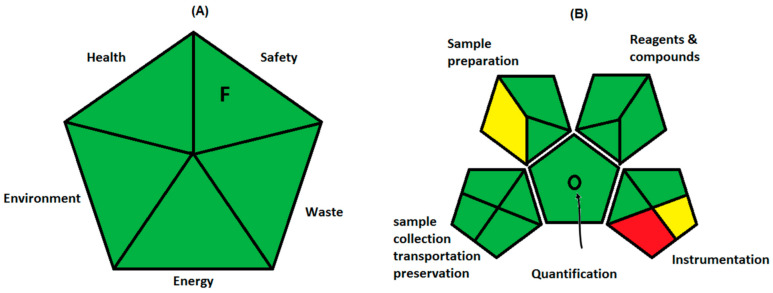
Greenness evaluation results for proposed analytical methods by Raynie et al. (**A**) and GAPI (**B**).

Three different greenness evaluation techniques were used to estimate the ecofriendly nature of the UV spectroscopic methods developed in this study. First method is semi qualitative method developed by Raynie et al. [41]. Ethanol was used as a solvent to prepare the stock solution of one of the analytes and further dilutions were made with water. Ethanol [42] and water are safe solvents in terms of health, safety, and environment hazards. The instrument used was spectrophotometer, and the evaporation of ethanol was negligible; hence, the energy utilized by these methods is safe. Further, each sample prepared contains less than 10% ethanol along with micrograms of analytes, and hence, the waste generated is less than 50 g. Hence, the green assessment profile (Figure 6A) of the proposed derivative UV spectrophotometry is completely ecofriendly (Figure S5). 

According to the analytical eco-scale method [43] penalty points (pp) were calculated and subtracted from 100 to determine the eco-scale of the method (Table S1). The eco-scale score above 75 for the analytical method is considered to be ecofriendly. The total of penalty points for the proposed methods was 6 (waste volume pp 3 (1 to 10 mL) and no treatment for waste pp 3). Hence, the eco-scale score for the developed derivative spectroscopic methods was 94, indicating the greenness of the methods. 

Plotka-Wasylka introduced the combination of qualitative and quantitative greenness calculation method known as green analytical procedure index (GAPI) [44]. GAPI could be applied for different analytical procedures by considering the 15 different parameters, starting from sample preparation, volume and health hazards of solvents and reagents, instrumentation and waste quantity and its processing (Table S2). In the developed spectroscopic methods, there is inline sample preparation; however, no storage and transportation was required. In addition, no extraction in the sample preparation (direct method) and green solvents were used for sample preparation without any additional treatments. Less than 10% ethanol was solvents, having irritant NFPA and flammability score 1 with no special hazard. The volume of waste is 1 to 10 mL per sample and no waste treatment; hence, sections 14 and 15 were colored yellow and red, respectively. However, overall results of GAPI as depicted in Table 5 and Figure 6B, indicated the greenness of the methods. Furthermore, the samples waste treatment needs to be developed to avoid the contamination of nature with the samples. 

## 4. Conclusions

Three simple, accurate, UV spectroscopic methods were established for concurrent quantification of REM and MET without the use of any complicated mathematical processing. Derivative and ratio derivative methods have many advantages over liquid chromatographic methods in terms of speed, economy, and specificity. Furthermore, the developed methods utilized water as a major solvent, making them economical and ecofriendly, which was confirmed by three greenness evaluation methods of analytical procedure. The applicability of the current methods could be assessed by analyzing both analytes in laboratory prepared solutions in different ratios and in tablets with excellent accuracy and precision. Hence, these methods could be utilized for regular quality-control study of REM and MET formulations in pharmaceutical industries and clinical laboratories. 

## Figures and Tables

**Table 1:** Regression parameters values for remogliflozin (REM) and metformin (MET).

Parameters	First Derivative Spectroscopic Method		Ratio Absorbance Difference Method		Ratio first Derivative Method	
Drugs	REM	MET	REM	MET	REM	MET
Wavelength [nm]	233.0	252.2	277.8 248.6	242.4 221.2	288.4	251.7
Linearity Range [µg mL^−1^]	1.0–20	2.5–35	1.0–20	2.5–35	1.0–20	2.5–35
LOD [µg mL^−1^]	0.180	0.660	0.290	0.850	0.240	0.780
LOQ [µg mL^−1^	0.560	1.850	0.860	2.290	0.730	2.170
Slop [m]	0.021	0.022	1.821	0.919	0.156	0.123
Intercept [c]	0.006	0.004	0.516	0.032	0.046	0.024
Correlation Coefficient [r^2^]	0.9985	0.9989	0.9989	0.9991	0.9995	0.9998

LOD: Limit of detection; LOQ: Limit of quantification.

**Table 2:** Inter-day and Intra-day results for remogliflozin (REM) and metformin (MET) by three proposed methods.

Drug Name	Amount of Drug [µg mL^−1^]	Inter-Day			Intra-Day		
		Amount found Mean ^a^ [*n* = 3] ± SD	%RSD	%Recovery	Amount found Mean ^b^ [*n* = 9] ± SD	%RSD	%Recovery
**First derivative spectroscopic method**							
REM	2.50	2.47 ± 0.02	0.81	98.80	2.52 ± 0.03	1.19	100.8
	7.50	7.45 ± 0.09	1.21	99.33	7.36 ± 0.12	1.63	98.13
	12.5	12.6 ± 0.21	1.67	100.6	12.4 ± 0.16	1.29	99.12
	17.5	17.3 ± 0.32	1.85	98.91	17.4 ± 0.14	0.80	99.43
MET	4.00	3.94 ± 0.06	1.52	98.50	3.93 ± 0.04	1.02	98.25
	14.0	13.9 ± 0.13	0.94	99.21	14.2 ± 0.12	0.84	101.5
	24.0	24.3 ± 0.39	1.60	101.42	23.7 ± 0.24	1.01	98.71
	32.0	31.6 ± 0.58	1.83	98.78	31.9 ± 0.37	1.16	99.59
**Ratio difference absorption method**							
REM	2.50	2.48 ± 0.03	1.21	99.20	2.54 ± 0.02	0.79	101.6
	7.50	7.41 ± 0.07	0.94	98.80	7.41 ± 0.06	0.81	98.80
	12.5	12.6 ± 0.11	0.87	101.0	12.4 ± 0.13	1.05	99.44
	17.5	17.3 ± 0.09	0.52	98.80	17.3 ± 0.21	1.21	99.03
MET	4.00	3.96 ± 0.04	1.01	99.00	3.90 ± 0.03	0.77	97.50
	14.0	14.1 ± 0.13	0.92	100.5	13.8 ± 0.22	1.60	98.50
	24.0	23.8 ± 0.18	0.76	98.96	13.7 ± 0.21	1.54	56.92
	32.0	31.9 ± 0.29	0.91	99.63	31.7 ± 0.29	0.91	99.16
**Ratio first derivative method**							
REM	2.50	2.46 ± 0.02	0.81	98.40	2.53 ± 0.01	0.40	101.2
	7.50	7.42 ± 0.06	0.81	98.93	7.46 ± 0.09	1.21	99.47
	12.5	12.4 ± 0.21	1.69	99.44	12.5 ± 0.17	1.36	99.76
	17.5	17.4 ± 0.22	1.27	99.31	17.4 ± 0.29	1.66	99.54
MET	4.00	3.97 ± 0.07	1.76	99.25	3.95 ± 0.03	0.76	98.75
	14.0	14.2 ± 0.12	0.84	101.5	14.1 ± 0.12	0.85	100.9
	24.0	23.8 ± 0.31	1.30	99.04	23.8 ± 0.33	1.39	99.04
	32.0	31.8 ± 0.48	1.51	99.47	31.8 ± 0.45	1.42	99.22

^a^ Average of three readings (*n* = 3), ^b^ Average of three readings (*n* = 9), RSD: Relative standard deviation, SD: Standard deviation.

**Table 3:** Application of the proposed method for quantification of REM and MET in laboratory prepared solution mixtures.

Ratio of REM:MET (µg mL^−1^)	Remogliflozin (% Recovery ± SD) ^a^			Metformin (% Recovery ± SD) ^a^		
	FDS	RAD	RFD	FDS	RAD	RFD
2:5	100.8 ± 0.01	99.57 ± 0.03	98.89 ± 0.02	98.50 ± 0.03	101.8 ± 0.02	98.36 ± 0.01
5:30	99.39 ± 0.04	98.79 ± 0.02	101.4 ± 0.02	98.10 ± 0.61	101.2 ± 0.51	101.3 ± 0.36
10:5	101.4 ± 0.21	98.91 ± 0.09	99.02 ± 0.03	98.71 ± 0.02	98.91 ± 0.03	100.7 ± 0.02
10:20	98.80 ± 0.12	99.08 ± 0.19	99.00 ± 0.12	101.7 ± 0.19	101.1 ± 0.07	99.04 ± 0.11
20:20	99.12 ± 0.18	101.0 ± 0.27	99.25 ± 0.13	100.8 ± 0.21	99.39 ± 0.17	101.3 ± 0.20
20:30	101.1 ± 0.07	101.3 ± 0.28	98.16 ± 0.22	98.55 ± 0.32	98.59 ± 0.49	98.67 ± 0.29

^a^ Average of three determinations. SD: Standard Deviations; FDS: First Derivative Spectroscopic method; RAD: Ratio Absorption Difference Method, RFD: Ratio First Derivative Method.

**Table 4:** Application of suggested methods for estimation of REM and MET in laboratory prepared tablets and use of standard addition technique.

Formulation	Drug	Amount of Drug [µg mL^−1^]	% Recovery		
			FDS	RAD	RFD
Prepared tablets (REM 100 mg +MET 500 mg)	REM	2.00	98.40	98.32	98.95
	MET	10.0	99.28	98.72	101.1
	REM	4.00	100.4	99.57	98.83
	MET	20.0	101.7	99.87	98.36
Prepared tablets (REM 100 mg +MET 1000 mg)	REM	2.00	98.87	101.9	101.0
	MET	20.0	101.4	99.80	98.87
	REM	3.00	99.00	99.55	98.25
	MET	30.0	98.60	98.80	99.50
**Standard addition method ^a^**					
REM added		1.00	101.1	99.19	98.75
		2.00	101.0	101.2	100.3
		3.00	99.09	99.04	98.83
		Across Mean	100.4	99.81	99.28
		% RSD	0.90	0.99	0.70
MET added		5.00	100.8	100.5	98.87
		10.0	99.74	99.88	99.50
		15.0	99.05	101.1	98.20
		Across Mean	99.87	100.5	98.86
		% RSD	0.72	0.48	0.53

^a^ An amount added to the previously analyzed sample (REM 2 µg mL^−1^ and MET 10 µg mL^−1^). FDS: First derivative spectroscopic method; RAD: ratio absorption difference method, RFD: ratio first derivative method; RSD: relative standard deviation.

**Table 5:** Green analytical procedure index parameter results for proposed methods.

Category	UV Spectroscopic Methods
Sample preparation	
Collection (1)	In-line
Preservation (2)	None
Transport (3)	None
Storage (4)	None
Type of method: direct or indirect (5)	Direct (no sample preparation)
Scale of extraction (6)	None
Solvents/reagents used (7)	Green Solvents
Additional treatments (8)	None
Reagent and solvents	
Amount (9)	<10 mL
Health hazard (10)	Ethanol, slightly toxic and irritant NFPA score 1
Safety hazard (11)	Ethanol, instability score 0, flammability score 1, no special hazard
Instrumentation	
Energy (12)	≤0.1 kWh per sample
Occupational hazard (13)	Hermetic sealing of analytical process
Waste (14)	1–10 mL
Waste treatment (15)	No treatment
QUANTIFICATION	Yes

## Data Availability

The data presented in this study is available in supplementary material.

## Supplementary Materials

The following are available online at https://www.mdpi.com/1660-4601/18/2/448/s1, Figure S1: Linearity curves for REM and MET by offered methods, Figure S2: Zero-order, first-order derivative, ratio, and ratio first-order derivative spectra of REM and MET for precision and accuracy by offered methods, Figure S3: Zero-order, first-order derivative, ratio, and ratio first-order derivative spectra of REM and MET for laboratory mixed solutions by offered methods, Figure S4: Zero-order, first-order derivative, ratio, and ratio first-order derivative spectra of REM and MET for formulation solutions by offered methods, Figure S5: Green assessment profile proposed by Raynie et al., Table S1: The penalty points (PPs) to calculate analytical eco-scale, Table S2: Green analytical procedure index parameters.
