# Supplementary material for: Development and Validation of Green UV Derivative Spectrophotometric Methods for Simultaneous Determination Metformin and Remogliflozin from Formulation: Evaluation of Greenness
Source: Int J Environ Res Public Health. 2021 Jan 8;18(2):448. doi: 10.3390/ijerph18020448 (PMC7827813; doi:10.3390/ijerph18020448)
Supplement: Supplementary file 1 [file ijerph-18-00448-s001.pdf]

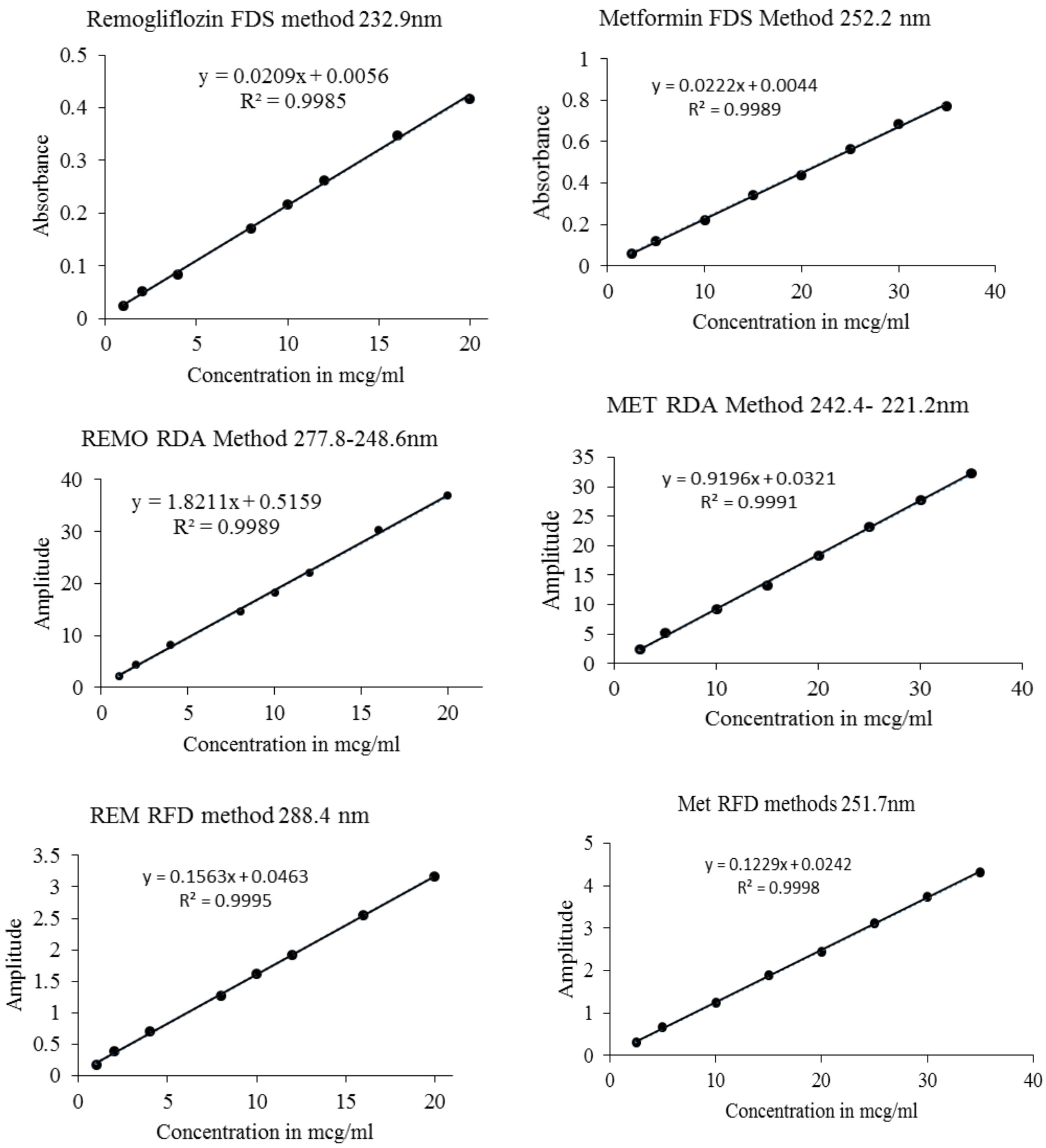

Figure S1 : Calibration curves for REM and MET by First derivative spectroscopic method (FDS), Ratio absorption derivative (RAD) and Ratio First derivative method (RFD)

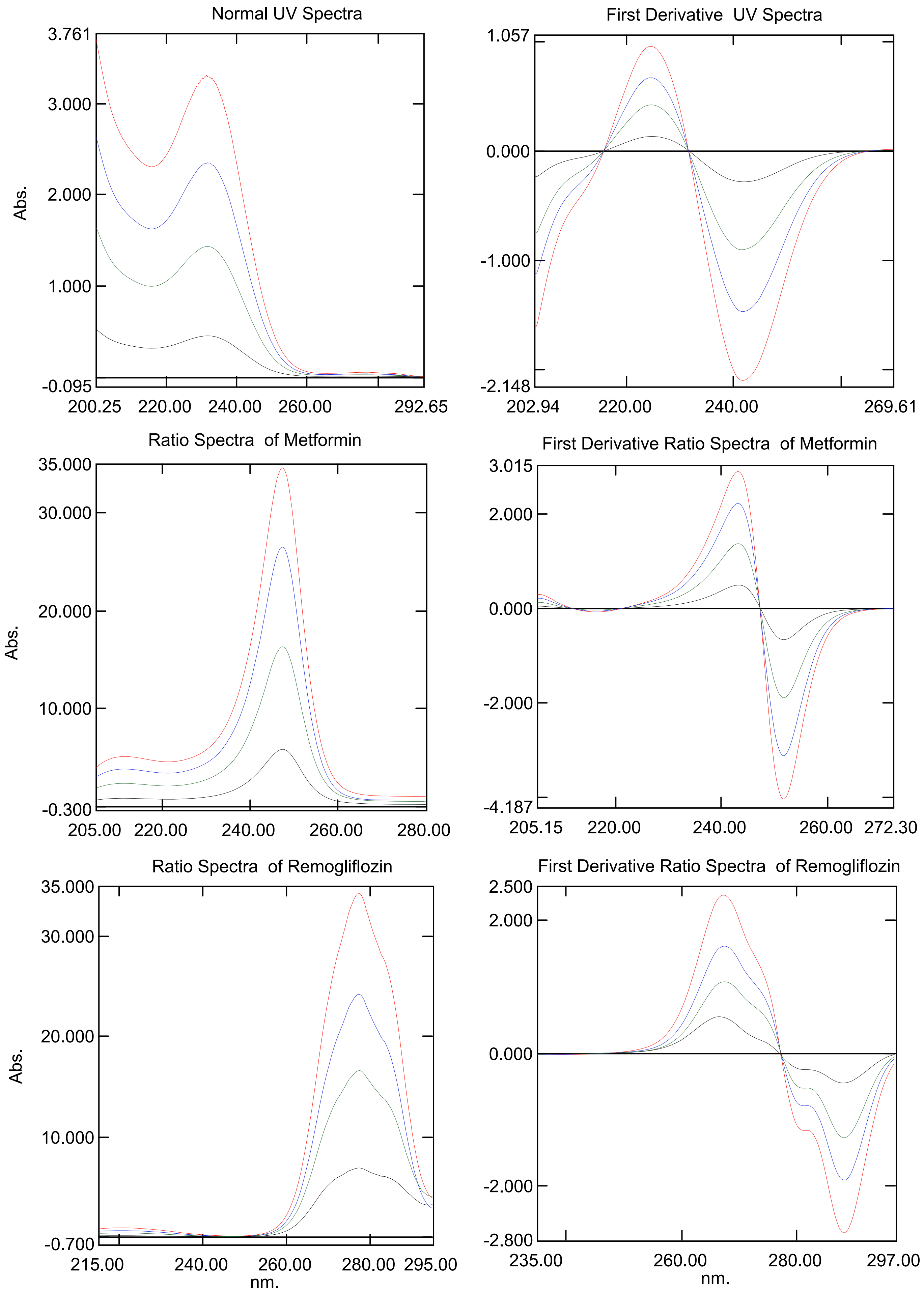

Figure S2: UV spectra of solutions consisting of 2.5, 7.5, 12.5, and 17.5 µg/ml of REM and 4, 14, 24, 32 µg/ml for accuracy and precision

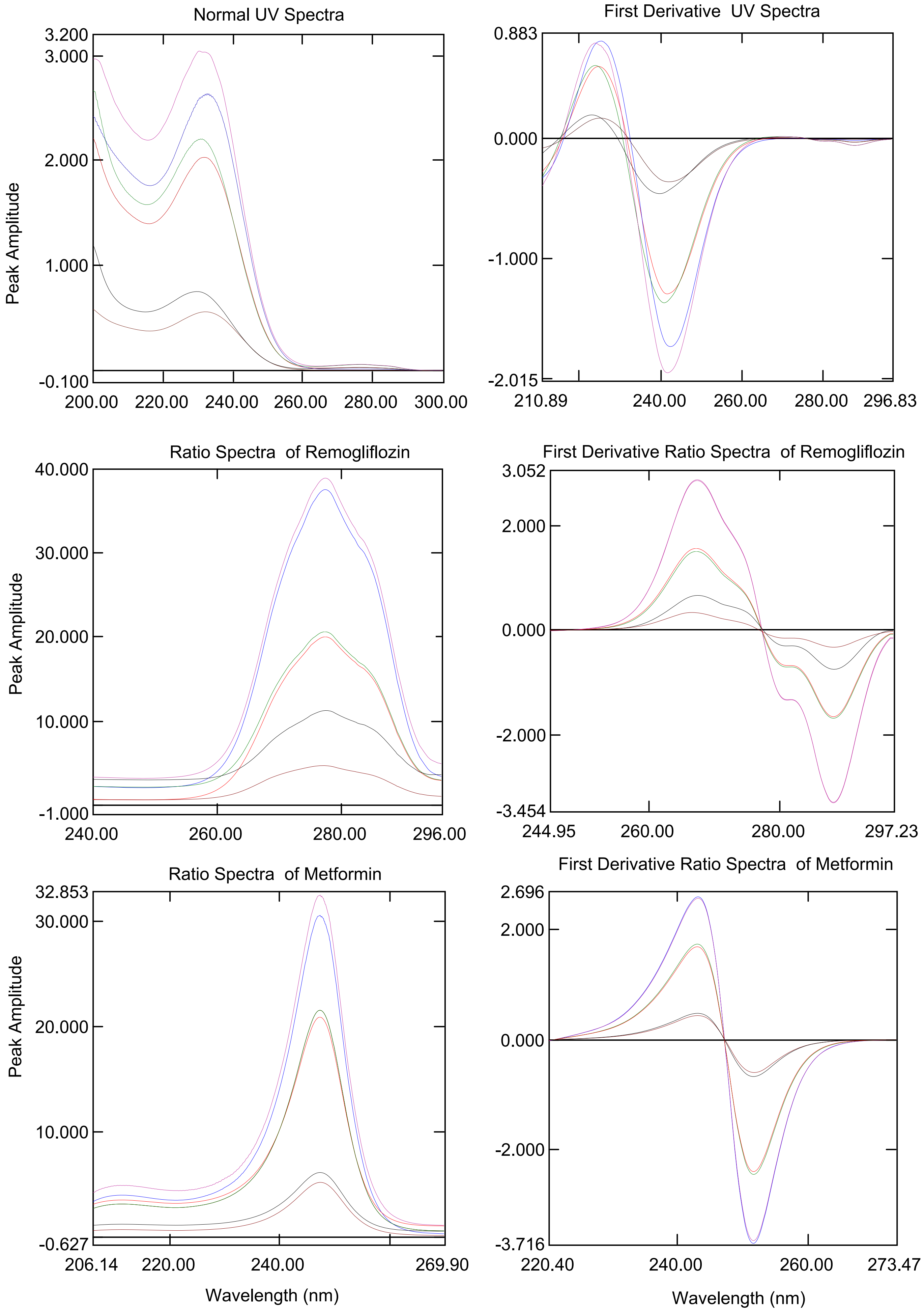

Figure S3: UV spectra of laboratory solutions consisting of REM:MET in ratio 2:5, 5:30, 10:5, 10:20, 20:20 and 20:30 µg/ml

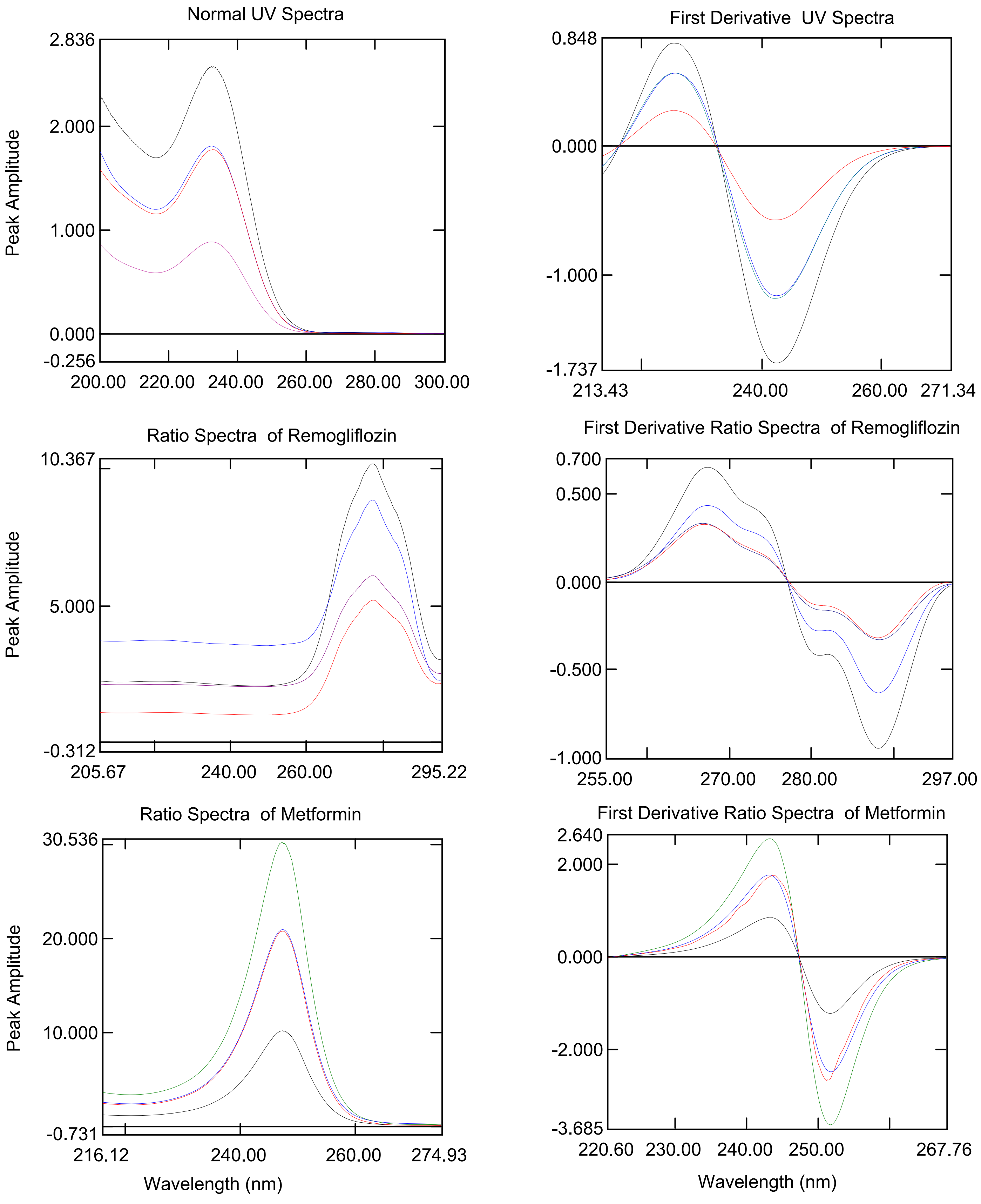

Figure S4: UV spectra of formulation solutions consisting of REM:MET (µg/mL) 2:10, 4:20, 2:20 and 3:30 µg/ml

Supplementary Material

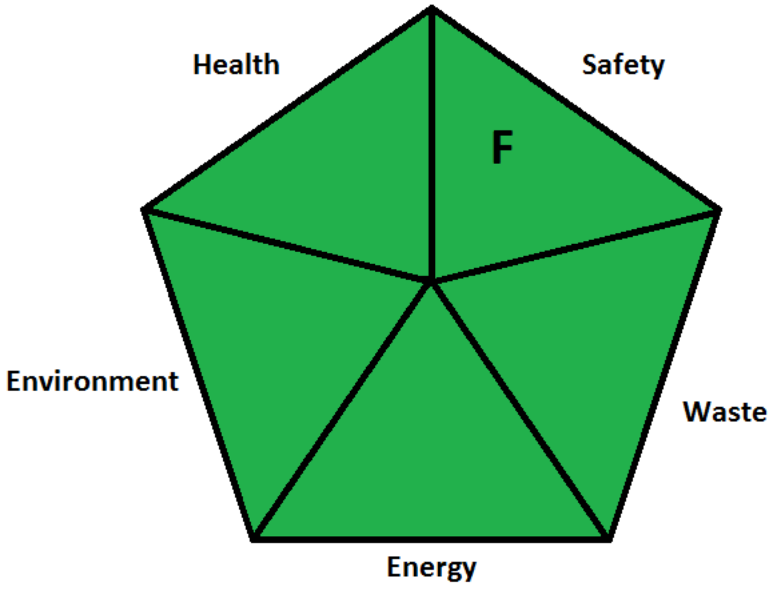

Category	Green	Yellow	Red
**Health Hazard**	Slightly toxic, slight irritant; NFPA health hazard score is 0 or 1.	Moderately toxic; could cause temporary incapacitation; NFPA = 2 or 3.	Serious injury on short term exposure; known or suspected small animal carcinogen; NFPA = 4.
**Safety Hazard**	Highest NFPA flammability, instability score of 0 or 1. No special hazards.	Highest NFPA flammability or instability score is 2 or 3, or a special hazard is used.	Highest NFPA flammability or instability score is 4.
**Environmental Hazard**	If less than 50 g of environmental hazards used.	If more than 50 g but less than 250 g used.	If more than 250 g used.
**Energy**	Wet chemistry method such as titration. Very little solvent evaporation.	Instrumental method such as GC, HPLC; moderate solvent evaporation.	Instrumental method such as GC-MS; high volume of solvent evaporated.
**Waste amount**	Total waste for processing one sample $\leq$ 50 g.	Total waste $\leq$ 250 g.	Total waste > 250 g.

Figure S5. Green assessment profile proposed by Raynie et al. [1]

References

D. Raynie, J. Driver, Green Assessment of Chemical Methods, In: 13th Annual Green Chemistry and Engineering Conference, *Maryland*, 2009.

Supplementary Material

Table S1. The penalty points (PPs) to calculate Analytical Eco-Scale [1]

Category	Sub-total PPs		Total PPs
	**Reagents**		
**Amount**	<10 mL (<10 g)	1	Amount PPs x hazard PPs
	10-100 mL (10-100 g)	2	
	>100 mL (>100 g)	3	
**Hazard**	None	0	
	Less severe hazard	1	
	More severe hazard	2	
	**Instruments**		
**Energy**	<0.1 kWh per sample		0
	<1.5 kWh per sample		1
	>1.5 kWh per sample		2
**Occupational hazard**	Hermetization of analytical proces		0
	Emission of vapours to the atmosphere		3
**Waste**	None		0
	<1 mL (<1 g)		1
	1-10 mL (1-10 g)		3
	>10 mL (>10 g)		5
	Recycling		0
	Degradation		1
	Pasivation		2
	No treatment		3

Table S2: Green analytical procedure Index parameters [2]

Category	Green	Yellow	Red
**Sample preparation**			
Collection (1)	In-line	On-line or at-line	Off-line
Preservation (2)	None	Chemical or physical	Physico-chemical
Transport (3)	None	Required	–
Storage (4)	None	Under normal conditions	Under special conditions
Type of method: direct or indirect (5)	No sample preparation	Simple procedures, eg. filtration, decantation	Extraction required
Scale of extraction (6)	Nano-extraction	Micro-extraction	Macro-extraction
Solvents/reagents used (7)	Solvent-free methods	Green solvents/reagents used	Non-green solvents/reagents used
Additional treatments (8)	None	Simple treatments (clean up, solvent removal, etc.)	Advanced treatments (derivatization, mineralization, etc.)
**Reagent and solvents**			
Amount (9)	< 10 mL (< 10 g)	10–100 mL (10–100 g)	> 100 mL (> 100 g)
Health hazard (10)	Slightly toxic, slight irritant; NFPA health hazard score = 0 or 1.	Moderately toxic; could cause temporary incapacitation; NFPA = 2 or 3.	Serious injury on short-term exposure; known or suspected small animal carcinogen; NFPA = 4.
Safety hazard (11)	Highest NFPA flammability or instability score of 0 or 1. No special hazards.	Highest NFPA flammability or instability score of2 or 3, or a special hazard is used.	Highest NFPA flammability or instability score of 4.
**Instrumentation**			
Energy (12)	≤0.1 kWh per sample	≤1.5 kWh per sample	> 1.5 kWh per sample
Occupational hazard (13)	Hermetic sealing of analytical process	–	Emission of vapours to the atmosphere
Waste (14)	< 1 mL (< 1 g)	1–10 mL (1–10 g)	> 10 mL (< 10 g)
Waste treatment (15)	Recycling	Degradation, passivation	No treatment
**ADDITIONAL MARK: QUANTIFICATION**			
Circle in the middle of GAPI: *Procedure for qualification and quantification*		No circle in the middle of GAPI: *Procedure only for qualification*	
NFPA: National Fire Protection Association			

References

1 Van Aken, K.; Strekowski, L.; Patiny, L. EcoScale, A semi-quantitative tool to select an organic preparation based on economical and ecological parameters. *Beilstein J. Org. Chem.* **2006**, *2*, 1–7.

2 Płotka-Wasylka, J. A new tool for the evaluation of the analytical procedure: Green Analytical Procedure Index. *Talanta* **2018**, *181*, 204–209.
